# Supplementary material for: The observational EURACAN prospective clinical registry dedicated to epithelioid hemangioendothelioma: The protocol of an international and collaborative effort on an ultra-rare entity
Source: PLoS One. 2024 Aug 12;19(8):e0308387. doi: 10.1371/journal.pone.0308387 (PMC11318912; doi:10.1371/journal.pone.0308387)
Supplement: Attachment — Submitted filename: Response Letter PlosOne_March_17.docx [file pone.0308387.s001.docx]

**RESPONSE LETTER**

**Reviewer #1, comments to the authors**: *The study represents an observational registry-based cohort study which includes new pathological and molecularly confirmed diagnosis of EHE, starting from the 1st December 2023, with an estimated completion date of December 2033. The study represents the first effort of this kind, being joined by 21 sarcoma reference centers across EU and UK, spanning 10 countries. It has a projected combined accrual of 100 cases/tear. The goal is to collect full details on patients and disease features, treatment and outcome, according to common clinical practice guidelines developed and shared with all the contributing centers. This effort is highly laudable and will be the first STARTER international registry, fully dedicated to an ultra-rare sarcoma such as EHE, which will serve as an example for other sarcomas.*

**One point that the authors may want to clarify or add relates to the semi-annual updates – if one particular systemic regimen has shown encouraging results in few patients – is there a mechanism in place to inform the other contributors? Meaning, can this registry serve as an interactive/active platform and have a feedback mechanism for the data analysis, not only at the end of the 10 years of observation.**

Thanks to the reviewer for pointing this out. Actually, the 10-year span was meant only to provide a tentative length of the registry. Within the registry, yearly meeting will be scheduled with all the contributing centers and relevant stakeholders in order to discuss together the progresses and challenges of the registry, and to share observations, especially if useful for clinical practice, which may arise from analyses performed. It is envisaged, in fact, to carry out the analyses on a yearly basis and in a scalable way based on the number of cases registered and the years of follow-up accrued (i.e. starting from descriptive analyses to move on to predictive and prognostic analyses as the years of follow-up and the number of patients increases). The following sentence has been added at page 14, paragraph “Status and timeline of the registry” in order to improve clarity: “In order to promptly share with all the contributing centres and relevant stakeholders the progresses done by the EHE registry, the challenges encountered and observation derived from the analyses of patients included in the registry, especially if useful for clinical practice, yearly update meeting will be scheduled”.

**Second point, a mandatory 6-monthly update is requested from the participating sites. Why only 6 month and not 1 year – as most EHE have an indolent behavior, with some multifocal liver or even lung EHE, showing no changes for years.**

The optimal length of the mandatory data update (which is requested in absence of events) was extensively discussed also with all the sarcoma reference centers joining this project. A 6-month interval was selected essentially for 3 reasons:

1- if it is true that, within EHE, some cases are indolent and characterized by prolonged, spontaneous disease stability, some others can slowly progress overtime or even show a very aggressive behavior. Describing the evolution of the disease in progressive cases and how this might change with treatment start is one of the goals of the registry, and we felt that this might be better and more reliably done with a 6-month interval,

2- the compulsory follow-up it is not only meant to provide data on disease status and survival, but also to monitor changes overtime in clinical features (including signs, symptoms, fertility, concomitant medications) which might influence EHE evolution and play a role as prognostic factors,

3- it was felt that a 6-monthly follow up could represent a reasonable interval in order to minimize the impact of different follow-up schedules across contributing centers in reported progression-free survival.

**There are many EHE registry objectives listed. However, among prognostic and predictive factors will be important to add under the molecular features: NGS findings when available, such as secondary hits: CDKN2A/B deletion. TERT promoter mutations, etc.**

We totally agree with the reviewer on the importance of capturing these data. The registry, for its own nature, does not foresee the collection of detailed molecular data outside standard practice, nor the storage of pathological samples and / or imaging. Still, the registry captures the availability, at the contributing institution, of pathological samples (baseline and recurrences), NGS data and imaging, in order to facilitate patients’ identification future dedicated studies which will imply the use of these data. The following sentence has been added at page 8, paragraph “Data collection”: “Although the EHE registry per se does not foresee any collection of pathological samples and / or imaging, the availability for each enrolled patient of pathological samples, massive parallel sequencing data and imaging at the contributing institution will be captured by the registry, in order to facilitate patients’ identification for future dedicated studies which will imply the use of these data”.

**Minor comments: mostly rephrasing and typos**

**- ‘peculiar molecular and pathologic features’ – replace ‘peculiar’ with ‘distinctive’ – in the Abstract intro as well as from the 1st sentence in the Introduction**

**- delete word ‘extremely’ before variable clinical behavior in the Abstract intro**

**- fix typo: ‘Centres’ with ‘centers’ in abstract settings, 2nd page intro and discussion**

**Intro: use italics for gene names and update nomenclature for gene fusions ‘-‘ replace with ‘::’**

Thanks for spotting, done as requested.
